# Neurokinin 1 receptor-expressing projection neurons in laminae III and IV of the rat spinal cord have synaptic AMPA receptors that contain GluR2, GluR3 and GluR4 subunits

**DOI:** 10.1111/j.1460-9568.2009.06633.x

**Published:** 2009-02

**Authors:** Andrew J Todd, Erika Polgár, Christine Watt, Mark ES Bailey, Masahiko Watanabe

**Affiliations:** 1 Neuroscience and Molecular Pharmacology, Faculty of Biomedical and Life Sciences, University of GlasgowGlasgow G12 8QQ, UK; 2 Molecular Genetics, Faculty of Biomedical and Life Sciences, University of GlasgowGlasgow G12 8QQ, UK; 3 Department of Anatomy, Hokkaido University School of MedicineSapporo 060-8638, Japan

**Keywords:** antigen retrieval, GluR1, glutamate, NK1 receptor, projection neuron

## Abstract

α-Amino-3-hydroxy-5-methyl-4-isoxazolepropionic acid receptors (AMPArs), which mediate fast excitatory glutamatergic transmission, are tetramers made from four subunits (GluR1–4 or GluRA–D). Although synaptic AMPArs are not normally detected by immunocytochemistry in perfusion-fixed tissue, they can be revealed by using antigen retrieval with pepsin. All AMPAr-positive synapses in spinal cord are thought to contain GluR2, while the other subunits have specific laminar distributions. GluR4 can be alternatively spliced such that it has a long or short cytoplasmic tail. We have reported that <10% of AMPAr-containing synapses in lamina II have the long form of GluR4, and that these are often arranged in dorsoventrally orientated clusters. In this study, we test the hypothesis that GluR4-containing receptors are associated with dorsal dendrites of projection neurons in laminae III and IV that express the neurokinin 1 receptor (NK1r). Immunostaining for NK1r was carried out before antigen retrieval, and sections were then reacted to reveal GluR2 and either GluR4 (long form), GluR3 or GluR1. All NK1r-positive lamina III/IV neurons had numerous GluR2-immunoreactive puncta in their dendritic plasma membranes, and virtually all (97%) of the puncta tested were labelled (usually strongly) with the GluR4 antibody. Sizes of puncta varied, but many were elongated and they were significantly larger than nearby puncta that were not associated with the NK1r cells. None of the GluR2 puncta on these cells was positive for GluR1, while 85% were GluR3-immunoreactive. These results show that synaptic AMPArs on the dendrites of the lamina III/IV NK1r projection neurons contain GluR2, GluR3 and GluR4, but not GluR1 subunits.

## Introduction

The spinal dorsal horn can be divided into six laminae based on cytoarchitectonic criteria (Rexed, 1952). Its primary afferent input is arranged in an orderly manner, with fine (mainly nociceptive) axons terminating in laminae I–II, and large (predominantly low-threshold mechanoreceptive) axons arborizing in a region that extends ventrally from inner lamina II (lamina Iii; Todd & Koerber, 2006). Although most dorsal horn neurons have axons that remain within the spinal cord (interneurons), there are discrete groups of cells with axons that project to the brain (projection neurons). One population of projection neurons consists of large cells with somata in lamina III–IV that express the neurokinin 1 receptor (NK1r, the main receptor for substance P) and have dorsal dendrites that enter lamina I (Bleazard *et al.*, 1994; Liu *et al.*, 1994; Brown *et al.*, 1995; Littlewood *et al.*, 1995; Marshall *et al.*, 1996; Todd *et al.*, 2000).

Glutamate, the main excitatory neurotransmitter in the spinal cord, is used by primary afferents, excitatory interneurons and projection neurons (Broman *et al.*, 1993; Todd *et al.*, 2003; Maxwell *et al.*, 2007). Glutamate released at synapses formed by axons of these cells acts on α-amino-3-hydroxy-5-methyl-4-isoxazolepropionic acid receptors (AMPArs), which mediate fast synaptic transmission (Yoshimura & Jessell, 1990; Yoshimura & Nishi, 1992) and play a major role in perception of both acute and chronic pain (Dickenson *et al.*, 1997; Garry & Fleetwood-Walker, 2004). AMPArs are tetramers made from four subunits (GluR1–4/GluRA–D), and subunit composition affects receptor properties, for example receptors lacking GluR2 are Ca^2+^-permeable (Burnashev *et al.*, 1992).

Synaptic AMPArs are generally not revealed with conventional immunocytochemistry, due to cross-linking of synaptic proteins following aldehyde fixation. However, they can be detected after antigen retrieval with pepsin (Watanabe *et al.*, 1998), and we have used this approach to demonstrate the distribution of each of the subunits at glutamatergic synapses in rat spinal cord (Nagy *et al.*, 2004; Polgár *et al.*, 2008a). We showed that GluR2 was present at virtually all AMPAr-containing puncta (which correspond to glutamatergic synapses) throughout the grey matter, and that although GluR1 and GluR3 were each present at ∼60% of puncta in laminae I–II, staining with antibody against the C-terminal portion of GluR4 was restricted to 23% of AMPAr-positive puncta in lamina I and <10% of those in lamina II (Polgár *et al.*, 2008a). The GluR4 subunit can be alternatively spliced, such that it has a long or short C-terminal tail (Gallo *et al.*, 1992), and the C-terminal antibody used by Polgár *et al.* only detects the long form. GluR4-immunoreactive puncta in laminae II–III were often arranged in dorsoventrally orientated rows, and we suggested that these corresponded to synapses on dendrites of lamina III/IV NK1r-immunoreactive projection neurons (Polgár *et al.*, 2008a). However, lamina II contains dorsally directed dendrites belonging to other neurons (Polgár *et al.*, 2007a, b), as well as ventral dendrites of some lamina I projection cells (Marshall *et al.*, 1996). The aim of this study was therefore to test the hypothesis that lamina III/IV NK1r-expressing projection neurons have synaptic AMPArs containing the long form of GluR4.

## Materials and methods

### Animals, tissue processing and immunocytochemistry

All experiments were approved by the Ethical Review Process Applications Panel of the University of Glasgow, and were performed in accordance with the UK Animals (Scientific Procedures) Act 1986.

Sixteen adult male Wistar rats (190–370 g; Harlan, Loughborough, UK) were deeply anaesthetized with pentobarbitone and perfused through the left ventricle with a fixative consisting of 4% freshly depolymerized formaldehyde. Lumbar spinal cord segments were removed and stored in the same fixative for 4–8 h at 4°C, before being cut into parasagittal 60-μm-thick sections with a Vibratome. Sections were immersed in 50% ethanol for 30 min prior to immunoreaction to enhance antibody penetration (Llewellyn-Smith & Minson, 1992).

In preliminary experiments it was found that pepsin treatment, which was needed to reveal synaptic AMPAr subunits, prevented immunostaining with NK1r antibodies. We therefore carried out an initial reaction for the NK1r with a tyramide signal amplification (TSA) method, as we have found that this preserves immunofluorescence staining during subsequent antigen retrieval with pepsin (Nagy *et al.*, 2004). To examine the expression of synaptic AMPAr subunits on the lamina III/IV NK1r-immunoreactive neurons, sections were initially incubated for 3 days in rabbit antibody against the NK1 receptor (Sigma, Poole, UK; cat number S8305; 1 : 25 000–400 000), and then processed with a TSA kit (tetramethylrhodamine; PerkinElmer Life Sciences, Boston, MA, USA) according to the manufacturer’s instructions. The sections were rinsed and treated with pepsin (Dako, Glostrup, Denmark; 0.5–1.0 mg/mL for 10 min at 37°C) as described previously (Watanabe *et al.*, 1998; Nagy *et al.*, 2004). They were incubated for 72 h in mouse monoclonal antibody against GluR2 (Chemicon, Chandlers Ford, UK; Clone 6C4; 1 : 300) together with one of the following polyclonal antibodies: (a) rabbit anti-GluR1 (Chemicon; cat number AB1504; 1 : 300); (b) goat anti-GluR3 (Polgár *et al.*, 2008a; 1 : 300–500); or (c) rabbit anti-GluR4 (LabVision Products, Runcorn, Cheshire, UK; cat number RB-9059; 1 : 100). They were then incubated overnight in species-specific secondary antibodies raised in donkey and conjugated to Alexa 488 (Invitrogen, Paisley, UK; 1 : 500) or Cy5 (Jackson Immunoresearch, West Grove, PA, USA; 1 : 100), mounted in anti-fade medium and stored at −20°C.

Formaldehyde-fixed lumbar spinal cord sections from two mice in which the GluR4 gene was knocked out (*Gria4*^*tm1Dgen*^; Beyer *et al.*, 2008) and from two wild-type littermates were used to test the specificity of the GluR4 antibody. Transverse Vibratome sections were treated with pepsin and incubated in mouse monoclonal antibody to GluR2 (1 : 300) together with rabbit antibody against GluR4 (1 : 100). They were then incubated overnight in species-specific secondary antibodies raised in donkey and conjugated to Alexa 488 (Invitrogen, Paisley, UK; 1 : 500) or Cy5 (Jackson Immunoresearch, West Grove, PA, USA; 1 : 100) and mounted in anti-fade medium.

The NK1r antibody was raised against a peptide corresponding to amino acids 393–407 of the rat NK1r, and it has been shown that there is no staining with this antibody in CNS tissue from mice in which the NK1r has been deleted (NK1r−/−; Ptak *et al.*, 2002). The GluR2 antibody recognizes an N-terminal (extracellular) epitope, while the antibodies against GluR1 and GluR3 were raised against peptides corresponding to the C-terminal (intracellular) parts of the proteins (last 13 residues of the rat GluR1 subunit and residues 830–862 of the mouse GluR3 subunit). We have previously shown that the punctate staining for GluR1, GluR2 and GluR3 that is seen with these antibodies after antigen retrieval with pepsin is absent from mice in which the corresponding genes have been deleted (Polgár *et al.*, 2008a). The rabbit GluR4 antibody was raised against a peptide from the C-terminus of human GluR4, which corresponds to the C-terminal region of the long form of rat GluR4 (Gallo *et al.*, 1992).

### Confocal microscopy and analysis

Fifteen NK1r-immunoreactive cells with somata located in lamina III or IV were selected from sections reacted with each of the AMPAr subunit antibody combinations (GluR2/GluR1, GluR2/GluR3, GluR2/GluR4). Because immunostaining is restricted to the superficial parts of the sections, cells were only chosen if part of their dendritic tree approached the section surface. The cells were scanned with a Bio-Rad Radiance 2100 confocal microscope with Argon, HeNe and red diode lasers. Scanning was carried out sequentially (to avoid fluorescent bleed-through) through a 60× oil-immersion lens with a z-step of 0.5 μm, and the resulting image stacks were analysed with Neurolucida for Confocal software (MBF, Colchester, VT, USA). For each cell, images representing NK1r and GluR2 were initially viewed. The cell body and dendritic tree were drawn, and the locations of all GluR2 puncta that were located within the cell membrane (identified by immunostaining for NK1r) were plotted onto these drawings. Only regions of cell body or dendritic tree that were close to the section surface were examined for GluR2 puncta, and only cells that had at least 20 GluR2 puncta in their dendritic and/or somatic membrane were included in the study. The files representing the other AMPAr subunit (GluR1, GluR3 or GluR4) were then examined, and the presence or absence of staining for these subunits at each of the selected GluR2 puncta on the cell was determined. The approximate position of the border between laminae II and III was identified with dark-field microscopy.

During the course of the analysis we observed that nearly all of the GluR2-containing puncta on the dendrites of the lamina III/IV NK1r cells were GluR4-positive (see below), and that the GluR4-immunostaining in these puncta was generally bright, compared with that in other puncta in the dorsal horn. However, as reported previously (Polgár *et al.*, 2008a), GluR4 was only observed in a minority of AMPAr-positive puncta within laminae I–III. We therefore compared the relative intensity of immunostaining for GluR4 vs. GluR2 in puncta on the lamina III/IV NK1r cells with that for GluR4-containing puncta in lamina IV that were not associated with NK1r-immunoreactive cells. Because the intensity of immunostaining for both subunits became weaker with increasing depth below the section surface, only puncta on the NK1r cells that were within 5 μm of this surface were selected, and only those cells (*n*=9) that had at least 15 puncta within this depth were analysed in this part of the study. For each of these cells, a set of scans for GluR2, GluR4 and NK1r was obtained through the dendrites of the cells and also from a region of lamina IV that lay immediately ventral to the soma. The confocal settings (laser power, confocal aperture and gain) were identical for each scan in a set, and care was taken to ensure that no pixels corresponding to GluR2 or GluR4 were saturated. The scans were analysed with metamorph software (Universal Imaging, Downington, PA, USA), and threshold values were set for GluR2 and GluR4. Thirty puncta were selected from lamina IV for each set by applying a grid to a single confocal image (located ∼2 μm below the section surface) and identifying those puncta nearest the bottom right-hand corner of the grid squares with at least eight contiguous pixels that exceeded threshold for either subunit and at least one pixel that was suprathrehold for each subunit. These puncta were then followed through the z-series to the optical section in which they were largest, and the region containing suprathreshold pixels on this optical section was outlined. The mean luminance value for pixels representing GluR2 and GluR4 within the outlined area was determined for each punctum. A similar method was used to determine the mean luminance values for GluR2 and GluR4 in the GluR4-immunoreactive puncta on the dendrites of the NK1r-immunoreactive neuron that were within 5 μm of the section surface. For each of the nine NK1r cells, the mean ratio for GluR4 : GluR2 pixel luminance values was then calculated for the puncta in its dendritic membranes and also for those puncta in lamina IV that were not associated with NK1r-immunoreactive cells, and these were normalized so that the ratio of GluR4 : GluR2 for lamina IV puncta was assigned a value of 1.

We also noted that the GluR4/GluR2 puncta on the lamina III/IV NK1r cells were often longer than nearby GluR2 puncta that were not associated with these cells. We therefore measured the lengths of all of the selected puncta that were GluR4-positive on the 15 cells examined with the NK1r/GluR2/GluR4 combination. These were then compared with the lengths of a randomly selected sample of 600 GluR2-positive/GluR4-negative puncta from laminae II and III in these scans that were not associated with NK1r-immunoreactive cells. For the latter sample, 30 puncta in lamina II and 30 in lamina III were selected from scans of 10 of the cells, by placing a grid on a confocal image and selecting the punctum closest to the lower right corner of each grid square that fulfilled these criteria. Each of these puncta was then followed to the optical section on which it was largest, and its length was measured. For the NK1r-immunoreactive lamina III/IV neurons we compared the lengths of the GluR4 puncta associated with dendrites in the superficial dorsal horn (laminae I–II) with those on dendrites in deeper laminae (III–V), and also looked for evidence that the size of puncta was correlated with distance from the soma.

## Results

### Appearance of immunostaining with NK1r and AMPAr subunit antibodies

As reported previously (Bleazard *et al.*, 1994; Liu *et al.*, 1994; Nakaya *et al.*, 1994; Brown *et al.*, 1995; Littlewood *et al.*, 1995; Mantyh *et al.*, 1997; Naim *et al.*, 1997, 1998; Todd *et al.*, 2000), large NK1r-immunoreactive neurons with cell bodies located in laminae III or IV and long dorsally directed dendrites that entered the superficial dorsal horn were frequently seen in parasagittal sections of rat spinal cord. Fifteen cells of this type that had dendrites close to the section surface were selected from sections reacted with each of the AMPAr subunit antibody combinations (GluR2/1, GluR2/3, GluR2/4). The depths of cell bodies below the dorsal white matter varied from 117 to 388 μm for the 45 selected neurons.

The distribution of AMPAr puncta was similar to that reported previously after antigen retrieval with pepsin (Nagy *et al.*, 2004; Polgár *et al.*, 2008a). Punctate staining for GluR2 was widespread throughout the dorsal horn, while staining for each of the other subunits showed a more restricted distribution. GluR1 puncta were most numerous in laminae I and II, and present at a lower density in lamina III. GluR3 puncta were found throughout the dorsal horn, although those in laminae I and II were often weakly stained compared with those in deeper laminae. GluR4 puncta were sparse in the superficial dorsal horn and became more numerous in deeper laminae (III–V). Although this was not formally analysed in this study, puncta that were immunoreactive with GluR1, GluR3 or GluR4 antibodies were virtually all GluR2-immunoreactive. Penetration of immunostaining with the AMPAr subunit antibodies was variable, but generally did not extend more than ∼10 μm into the section.

**Fig. 1:**
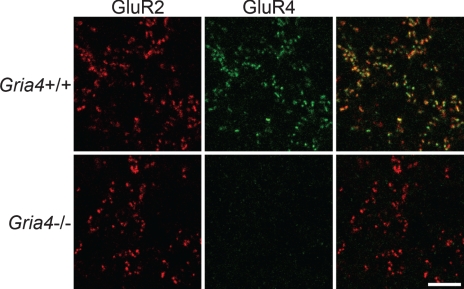
Staining for GluR2 and GluR4 in spinal cord sections from GluR4 knockout (*Gria4−/−*) and wild-type (*Gria4+/+*) mice. These confocal images show parts of the ventral horn from sections that had been reacted with antibodies against GluR2 and GluR4 after antigen retrieval with pepsin. Punctate staining for GluR2, which represents synaptic active zones at glutamatergic synapses, is visible in both cases. In the wild-type mouse, there is also punctate staining for GluR4, and this is extensively colocalized with the GluR2 staining. GluR4-immunoreactivity is absent from the knockout tissue. Both sets of images are projections of five optical sections at 0.3 μm z-separation. Scale bar: 5 μm.

In sections of spinal cord from the GluR4 knockout mice (*Gria4*^*tm1Dgen*^), punctate staining for GluR4 was absent, while staining for GluR2 appeared normal (Fig. 1). Both types of punctate staining were seen in spinal cord sections from the wild-type mice.

### AMPAr-subunit puncta on lamina III/IV NK1r cells

**Fig. 2:**
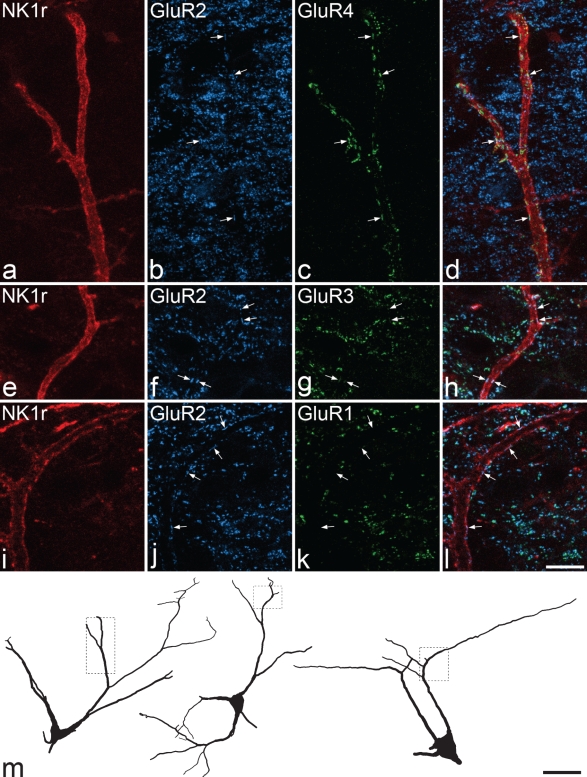
AMPAr subunit-immunoreactive puncta on dendrites of lamina III/IV neurokinin 1 receptor (NK1r)-expressing cells. (a–l) Projected confocal images obtained from sections stained for GluR2 and either GluR4 (a–d), GluR3 (e–h) or GluR1 (i–l). In each case, the section was initially reacted with anti-NK1r (TSA method), and then underwent pepsin treatment and immunoreaction for the corresponding AMPAr subunits. Parts of the dendritic trees of three neurons are shown in red (a, e, i). In each case, there are several GluR2-immunoreactive puncta that appear to be located within the dendritic membrane (b, d, f, h, j, l), and some of these are indicated with arrows. (a–d) The GluR2 puncta on this dendrite are also immunoreactive for GluR4. Although there are numerous GluR2 puncta throughout this field, there are very few GluR4-positive puncta, other than those associated with the dendrite of the NK1r-immunoreactive cell. (e–g) GluR2 puncta on this dendrite show very weak GluR3-immunoreactivity, compared with many of the surrounding puncta. (i–l) Although many of the GluR2-positive puncta in this field are GluR1-immunoreactive, those on the dendrite are not. (m) Drawings of the three cells that show the locations of the regions of dendrites illustrated in (a–l). The confocal images are projections of nine (a–d), four (e–h) or three (i–l) optical sections at 0.5 μm z-separation. Scale bars: 10 μm (a–l) and 50 μm (m).

All of the 45 NK1r-positive lamina III/IV cells had numerous GluR2-immunoreactive puncta that were judged to be in the dendritic plasma membrane, based on their alignment with the NK1r-immunostaining that outlined this membrane (Fig. 2d, h and l). Due to the limited penetration of AMPAr staining, puncta were only seen on regions of dendrite that were close to the section surface. These puncta were often elongated along the long axis of the dendritic shafts.

In the sections reacted with GluR4 and GluR2 antibodies, between 21 and 113 GluR2 puncta were identified on each of the 15 selected cells, and between 92% and 100% (mean 97%) of these puncta were also GluR4-immunoreactive (Fig. 2a–d). The few puncta on these cells that lacked GluR4 were located relatively deep within the section, and the absence of GluR4 staining may represent a false-negative result. Of the 799 GluR2 puncta identified on these cells, 392 were located in laminae I and II, and 407 in laminae III–V (395 on dendrites and 12 on cell bodies). The proportion of these that were GluR4-positive was 97% for each region. Although there were numerous GluR2-immunoreactive puncta in the neuropil surrounding the dendrites of the NK1r-positive cells, very few of those in the superficial dorsal horn were labelled with the GluR4 antibody (Fig. 2b–d). The GluR4-immunoreactivity in puncta associated with the NK1r-positive cells was generally strong compared with that of other GluR4 puncta in the deep dorsal horn that were seen in these sections, and to quantify this we compared the ratio of pixel luminance values for GluR4 : GluR2 between these populations (see Materials and methods). The normalized ratios of pixel luminance values for GluR4 : GluR2 in puncta on the nine NK1r cells that were analysed in this part of the study ranged from 1.71 to 3.69 (mean 2.87), and these values were significantly different from 1 (the normalized ratio of GluR4 : GluR2 on the lamina IV puncta that were not associated with NK1r cells; one-sample *t*-test, *P*<0.001).

**Fig. 3:**
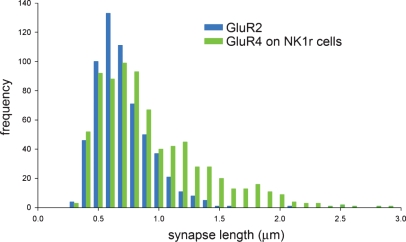
Sizes of AMPAr-positive puncta on the neurokinin 1 receptor (NK1r)-immunoreactive lamina III/IV cells compared with the sizes of other puncta in laminae II and III. The histogram shows the frequency distribution for the GluR4-positive puncta that were identified on the lamina III/IV NK1r-immunoreactive cells (green bars, *n*=775 puncta on 15 cells), and of GluR2-positive/GluR4-negative puncta in laminae II and III that were not associated with NK1r-immunoreactive dendrites (blue bars, *n*=600 puncta). For further details, see text.

The lengths of the 775 GluR4-immunoreactive puncta that were identified on these cells ranged from 0.26 to 2.89 μm (median 0.77 μm), while those of the 600 GluR2-positive/GluR4-negative puncta not associated with NK1r-immunoreactive cells varied from 0.26 to 2.06 (median 0.61 μm; Fig. 3). These values were significantly different (Mann–Whitney Rank Sum test, *P*<0.001).

**Fig. 4:**
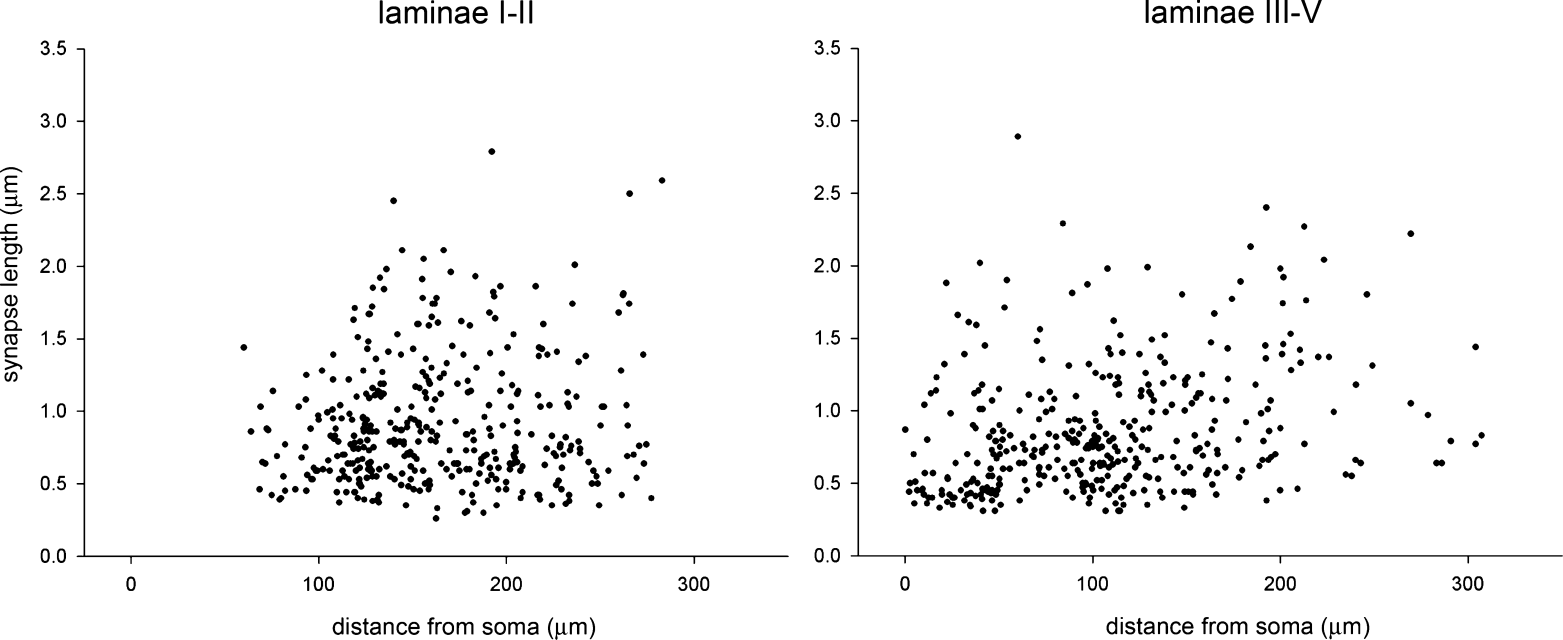
Scatter plots showing the relation between the size of GluR4 puncta on the dendrites of the NK1r-immunoreactive lamina III/IV cells and their distance from the soma. Puncta on dendrites in the superficial (I–II) and deep (III–V) laminae are plotted separately.

Comparison of the lengths of GluR4-positive puncta on dendrites of the NK1r-immunoreactive lamina III/IV neurons in different regions showed that those in laminae I–II (median 0.8 μm, *n*=382) differed significantly from those in laminae III–V (median 0.75, *n*=381; Mann–Whitney Rank Sum test, *P*<0.02). We therefore analysed the relationship between punctum size and distance from the soma separately for these two regions (Fig. 4). We found a weak positive correlation between size and distance from soma for puncta in laminae III–V (*R*_S_ = 0.31, Spearman Rank Correlation, *P*<0.001), but no correlation for those in laminae I–II (*R*_S_ = 0.07, *P*>0.05).

In sections reacted to reveal GluR2 and GluR3, 1105 GluR2-immunoreactive puncta were identified on the 15 selected NK1r lamina III/IV cells (between 20 and 179 from each cell), and between 69% and 100% (mean 85%) of these were GluR3-immunoreactive. Of these puncta, 660 were present in laminae I–II and 445 in laminae III–V. The proportions that were GluR3-immunoreactive were 86% for laminae I–II and 83% for laminae III–V. However, the GluR3 staining in puncta on the dendrites of the NK1r cells was generally weak, compared with that in many nearby puncta that were not associated with these cells (Fig. 2e–h). Altogether, 690 GluR2-immunoreactive puncta (446 from laminae I–II, 244 from laminae III–V) were identified on the 15 cells selected from sections that had been reacted with GluR1 and GluR2 antibodies (between 27 and 49 per cell). None of these puncta was GluR1-immunoreactive, although many of the surrounding GluR2 puncta were positive for GluR1 (Fig. 2i–l).

## Discussion

The main finding of this study is that dendrites belonging to the large NK1r-expressing projection neurons with cell bodies in laminae III and IV have AMPAr puncta that are strongly immunoreactive with a GluR4 antibody (which recognizes only the long form of this subunit), and also contain GluR2 and GluR3, but not GluR1. These cells therefore express the long form of GluR4, and their dorsal dendrites account for some of the clusters of GluR4-immunoreactive puncta that we have identified previously in laminae II and III (Polgár *et al.*, 2008a).

### Detection of synaptic AMPArs on NK1r-positive cells

There are several lines of evidence to support the suggestion that the punctate staining seen with antibodies against AMPAr subunits after antigen retrieval with pepsin represents receptors located at the postsynaptic aspect of glutamatergic synapses (Watanabe *et al.*, 1998). Glutamatergic axon terminals in the spinal cord can be identified by immunostaining for the vesicular glutamate transporters VGLUT1 and VGLUT2, or with markers for fine-diameter primary afferent terminals (antibodies against calcitonin gene-related peptide, CGRP, or binding of *Bandeiraea simplicifolia* isolectin B4, BSI-B4; Todd *et al.*, 2003; Oliveira *et al.*, 2003; Alvarez *et al.*, 2004). We have shown that punctate staining for AMPAr subunits is associated with each of these classes of axon terminal, and that for those types of axon that are known to make complex (glomerular) synaptic arrangements, boutons are often associated with several AMPAr puncta (Nagy *et al.*, 2004). We have also found that punctate staining with an antibody that recognizes all four AMPAr subunits is highly colocalized with that for the postsynaptic density protein PSD-95 (Polgár *et al.*, 2008a), and have used electron microscopy to demonstrate that immunostaining for GluR1 and GluR2 is located at the postsynaptic aspects of synapses (Nagy *et al.*, 2004). We had already confirmed the specificity of the antibodies against GluR1, GluR2 and GluR3 by demonstrating that the punctate staining seen with each of these after antigen retrieval was absent in spinal cord sections from the corresponding knockout mice (Polgár *et al.*, 2008a). Here we extend these observations by showing that punctate staining with the GluR4 antibody is not present in tissue from mice in which this subunit has been knocked out (Beyer *et al.*, 2008).

Although antigen retrieval with pepsin provides access for antibodies to receptors within the synaptic cleft and postsynaptic density, and thus allows identification of postsynaptic receptors in fixed tissue, we have found that it destroys several types of antigenicity, including that of the NK1r. We have previously demonstrated that this problem could be avoided by carrying out an initial TSA reaction to detect vulnerable antigens, and had used this approach to reveal the relationship between presynaptic markers (e.g. VGLUTs) and postsynaptic receptors (Nagy *et al.*, 2004). Here we show for the first time that this method can also be used with antibodies that stain the dendritic plasma membrane of specific neuronal populations in order to reveal synaptic receptors that are expressed by these cells. There is evidence that virtually all glutamatergic synapses in the dorsal horn contain the GluR2 subunit (Nagy *et al.*, 2004; Antal *et al.*, 2008), and we therefore used an antibody against this subunit to search for glutamatergic synapses on the lamina III/IV NK1r-immunoreactive neurons. Our finding that 97% of GluR2 puncta on these cells were GluR4-immunoreactive indicates that virtually all of their glutamatergic synapses contain the long form of GluR4. The great majority of GluR2 puncta on these cells were also labelled with the GluR3 antibody. However, the GluR3 labelling was generally weaker than that seen in many surrounding puncta, and this is consistent with our previous observation that puncta labelled with the GluR4 antibody in laminae II–III were either weakly labelled or unlabelled with anti-GluR3 (Polgár *et al.*, 2008a). In some cases the labelling for GluR3 in the puncta on these cells was only just above the level of detection, and GluR3 may therefore have been present at very low levels in the 15% of puncta that we classified as immunonegative.

### Glutamatergic synapses on NK1r-immunoreactive lamina III/IV NK1r cells

The presence of numerous AMPAr-containing puncta on the lamina III/IV NK1r-positive neurons indicates that they have a relatively high density of excitatory synapses on their dendritic shafts. It has been shown that these cells receive a strong synaptic input from substance P-containing primary afferent boutons (identified by the presence of both substance P and CGRP) on their dorsal dendrites (Naim *et al.*, 1997), and because most of the CGRP-immunoreactive boutons that contact these cells bind BSI-B4 (Sakamoto *et al.*, 1999) they presumably originate from unmyelinated (C) peptidergic primary afferents (Fang *et al.*, 2006). The size of GluR4-immunoreactive puncta on the dendrites of these cells was very variable, but many were highly elongated (>1 μm long). The peptidergic primary afferent terminals that are presynaptic to these cells are often very large, and are therefore likely to be associated with large synapses. Examples of synapses formed on the dendrites of these cells by substance P-containing primary afferents were illustrated in fig. 7 of Naim *et al.* (1997), and two of these were greater than 1.3 μm in length. It is therefore likely that many of the AMPAr puncta on the dorsal dendrites of these cells, including at least some of those >1 μm long, correspond to the synapses formed by substance P-containing primary afferents. These elongated synapses are likely to possess a relatively high number of AMPArs, and therefore to generate large excitatory postsynaptic currents when glutamate is released from the presynaptic terminal. All substance P-containing primary afferents are nociceptors (Lawson *et al.*, 1997), and the lamina III/IV NK1r cells have been shown to respond to various forms of noxious stimulation by upregulating the transcription factor Fos (Doyle & Hunt, 1999) and by phosphorylating extracellular signal-regulated kinases (Polgár *et al.*, 2007a).

Although these cells have very few contacts from other types of C fibre (Sakamoto *et al.*,1999), they do receive a significant synaptic input from myelinated (presumed low-threshold mechanoreceptive) afferents in laminae IIi and III (Naim *et al.*, 1998), and these presumably account for some of the AMPAr-containing puncta on their dendrites. We have also found that these cells have contacts from boutons with strong VGLUT2-immunoreactivity (E. Polgár and A.J. Todd, unpublished observations), and they are therefore likely to receive glutamatergic synaptic input from local excitatory interneurons (Oliveira *et al.*, 2003; Todd *et al.*, 2003; Alvarez *et al.*, 2004; Cheng *et al.*, 2004; Maxwell *et al.*, 2007; Schneider & Walker, 2007), and possibly also from descending axons that contain VGLUT2 (Todd *et al.*, 2003).

Although the sizes of GluR4 puncta on the dendrites of the lamina III/IV NK1r-immunoreactive neurons varied considerably in both deep and superficial regions of the dorsal horn, the median size for those in superficial laminae was significantly greater than that for those in deeper laminae. This is consistent with the suggestion that the larger synapses are associated with peptidergic primary afferents, as inputs from these afferents are more numerous on the dendrites of these cells that are located in the superficial dorsal horn than on dendrites in deeper laminae (Naim *et al.*, 1997). Two previous studies have reported a gradient of increasing size with greater distance from the soma for inhibitory synapses on the dendrites of teleost Mauthner cells (Triller *et al.*, 1990) and certain ventral horn interneurons in the rat (Alvarez *et al.*, 1997). This is thought to compensate for electrotonic attenuation of postsynaptic currents in distal dendrites. However, we observed only a weak correlation between size and distance from soma for AMPAr-containing puncta on dendrites in deeper laminae, and none for those in the superficial dorsal horn. The dendrites that were analysed in this study are considerably shorter than those of the ventral horn neurons examined by Alvarez *et al.* (1997; <300 μm compared with >1000 μm), and synapses on their distal portions will therefore be less affected by electrotonic attenuation. It is also possible that the poor correlation results from heterogeneity of excitatory synapses on the dendrites of these cells. If larger puncta (associated with peptidergic primary afferent terminals) are interspersed with smaller ones (originating from other sources), then this may mask a relationship between size and distance within each group.

The long form of GluR4 has certain features in common with GluR1. For example, both have long C-terminal tails and can be inserted into the postsynaptic density in response to synaptic activity by a mechanism that depends on protein kinase A (PKA) activation (Zhu *et al.*, 2000; Shi *et al.*, 2001; Esteban *et al.*, 2003). We have reported that the long form of GluR4 is present in relatively few synaptic puncta in the superficial dorsal horn (23% of those in lamina I and <10% of those in lamina II), and that it is not colocalized with GluR1 at synapses in lamina I (Polgár *et al.*, 2008a). We also reported that ∼65% of AMPAr puncta in laminae I and II contained GluR1. Taken together with the present results, this indicates that most (but not all) glutamatergic synapses in the superficial dorsal horn contain either GluR1 or the long form of GluR4, but that these subunits are generally not colocalized at synapses in this region. We have recently reported that a population of lamina I projection neurons that lack the NK1r and are characterized by a high density of gephyrin puncta (Puskár *et al.*, 2001) have synaptic AMPArs that contain both GluR3 and the long form of GluR4 (Polgár *et al.*, 2008b). We have also found that some of the large NK1r-expressing lamina I neurons have GluR4-containing puncta on their dendrites (A.J. Todd & E. Polgár, unpublished observations). This suggests that in the superficial dorsal horn synaptic AMPArs that contain the long form of GluR4 are largely restricted to the dendrites of projection neurons, with interneurons having synaptic receptors that contain various combinations of GluR1, GluR2 and GluR3.

It has been shown that there is phosphorylation of GluR1 at synapses in the superficial dorsal horn following noxious stimulation (Nagy *et al.*, 2004), and there is evidence that GluR1 subunits can be inserted into synapses in this region in various pain states (Galan *et al.*, 2004; Katano *et al.*, 2008; Larsson & Broman, 2008; Pezet *et al.*, 2008). It is therefore possible that phosphorylation of GluR4 and synaptic insertion of receptors that contain this subunit may contribute to the central sensitization of projection neurons in the superficial dorsal horn. Interestingly, although both GluR1- and GluR4-containing AMPArs can undergo PKA-mediated activity-dependent insertion, the insertion of GluR1-containing receptors also requires activation of calcium/calmodulin-dependent kinase II, while insertion of GluR4-containing receptors does not (Esteban *et al.*, 2003). It has been suggested that GluR1-containing AMPArs may therefore ‘have a higher threshold for plasticity’ than those that contain GluR4 (Esteban *et al.*, 2003). This may mean that in the dorsal horn AMPAr insertion into glutamatergic synapses on projection neurons occurs more readily than that into synapses on interneurons following noxious stimulation.

## Abbreviations

AMPAr: α-amino-3-hydroxy-5-methyl-4-isoxazolepropionic acid receptor
BSI-B4: *Bandeiraea simplicifolia* isolectin B4
CGRP: calcitonin gene-related peptide
NK1r: neurokinin 1 receptor
PKA: protein kinase A
TSA: tyramide signal amplification
VGLUT: vesicular glutamate transporter

## References

[b1] Alvarez FJ, Dewey DE, Harrington DA, Fyffe REW (1997). Cell-type specific organization of glycine receptor clusters in the mammalian spinal cord. J. Comp. Neurol..

[b2] Alvarez FJ, Villalba RM, Zerda R, Schneider SP (2004). Vesicular glutamate transporters in the spinal cord, with special reference to sensory primary afferent synapses. J. Comp. Neurol..

[b3] Antal M, Fukazawa Y, Eordogh M, Muszil D, Molnar E, Itakura M, Takahashi M, Shigemoto R (2008). Numbers, densities, and colocalization of AMPA- and NMDA-type glutamate receptors at individual synapses in the superficial spinal dorsal horn of rats. J. Neurosci..

[b4] Beyer B, Deleuze C, Letts VA, Mahaffey CL, Boumil RM, Lew TA, Huguenard JR, Frankel WN (2008). Absence seizures in C3H/HeJ and knockout mice caused by mutation of the AMPA receptor subunit Gria4. Hum. Mol. Genet..

[b5] Bleazard L, Hill RG, Morris R (1994). The correlation between the distribution of the NK1 receptor and the actions of tachykinin agonists in the dorsal horn of the rat indicates that substance P does not have a functional role on substantia gelatinosa (lamina II) neurons. J. Neurosci..

[b6] Broman J, Anderson S, Ottersen OP (1993). Enrichment of glutamate-like immunoreactivity in primary afferent terminals throughout the spinal cord dorsal horn. Eur. J. Neurosci..

[b7] Brown JL, Liu H, Maggio JE, Vigna SR, Mantyh PW, Basbaum AI (1995). Morphological characterization of substance P receptor-immunoreactive neurons in the rat spinal cord and trigeminal nucleus caudalis. J. Comp. Neurol..

[b8] Burnashev N, Monyer H, Seeburg PH, Sakmann B (1992). Divalent ion permeability of AMPA receptor channels is dominated by the edited form of a single subunit. Neuron.

[b9] Cheng L, Arata A, Mizuguchi R, Qian Y, Karunaratne A, Gray PA, Arata S, Shirasawa S, Bouchard M, Luo P, Chen CL, Busslinger M, Goulding M, Onimaru H, Ma Q (2004). Tlx3 and Tlx1 are post-mitotic selector genes determining glutamatergic over GABAergic cell fates. Nat. Neurosci..

[b10] Dickenson AH, Chapman V, Green GM (1997). The pharmacology of excitatory and inhibitory amino acid-mediated events in the transmission and modulation of pain in the spinal cord. Gen. Pharmacol..

[b11] Doyle CA, Hunt SP (1999). Substance P receptor (neurokinin-1)-expressing neurons in lamina I of the spinal cord encode for the intensity of noxious stimulation: a c-Fos study in rat. Neuroscience.

[b12] Esteban JA, Shi SH, Wilson C, Nuriya M, Huganir RL, Malinow R (2003). PKA phosphorylation of AMPA receptor subunits controls synaptic trafficking underlying plasticity. Nat. Neurosci..

[b13] Fang X, Djouhri L, McMullan S, Berry C, Waxman SG, Okuse K, Lawson SN (2006). Intense isolectin-B4 binding in rat dorsal root ganglion neurons distinguishes C-fiber nociceptors with broad action potentials and high Nav1.9 expression. J. Neurosci..

[b14] Galan A, Laird JM, Cervero F (2004). In vivo recruitment by painful stimuli of AMPA receptor subunits to the plasma membrane of spinal cord neurons. Pain.

[b15] Gallo V, Upson LM, Hayes WP, Vyklicky L, Winters CA, Buonanno A (1992). Molecular cloning and development analysis of a new glutamate receptor subunit isoform in cerebellum. J. Neurosci..

[b16] Garry EM, Fleetwood-Walker SM (2004). A new view on how AMPA receptors and their interacting proteins mediate neuropathic pain. Pain.

[b17] Katano T, Furue H, Okuda-Ashitaka E, Tagaya M, Watanabe M, Yoshimura M, Ito S (2008). N-ethylmaleimide-sensitive fusion protein (NSF) is involved in central sensitization in the spinal cord through GluR2 subunit composition switch after inflammation. Eur. J. Neurosci..

[b18] Larsson M, Broman J (2008). Translocation of GluR1-containing AMPA receptors to a spinal nociceptive synapse during acute noxious stimulation. J. Neurosci..

[b19] Lawson SN, Crepps BA, Perl ER (1997). Relationship of substance P to afferent characteristics of dorsal root ganglion neurones in guinea-pig. J. Physiol..

[b20] Littlewood NK, Todd AJ, Spike RC, Watt C, Shehab SA (1995). The types of neuron in spinal dorsal horn which possess neurokinin-1 receptors. Neuroscience.

[b21] Liu H, Brown JL, Jasmin L, Maggio JE, Vigna SR, Mantyh PW, Basbaum AI (1994). Synaptic relationship between substance P and the substance P receptor: light and electron microscopic characterization of the mismatch between neuropeptides and their receptors. Proc. Natl Acad. Sci. USA.

[b22] Llewellyn-Smith IJ, Minson JB (1992). Complete penetration of antibodies into vibratome sections after glutaraldehyde fixation and ethanol treatment: light and electron microscopy for neuropeptides. J. Histochem. Cytochem..

[b23] Mantyh PW, Rogers SD, Honore P, Allen BJ, Ghilardi JR, Li J, Daughters RS, Lappi DA, Wiley RG, Simone DA (1997). Inhibition of hyperalgesia by ablation of lamina I spinal neurons expressing the substance P receptor. Science.

[b24] Marshall GE, Shehab SA, Spike RC, Todd AJ (1996). Neurokinin-1 receptors on lumbar spinothalamic neurons in the rat. Neuroscience.

[b25] Maxwell DJ, Belle MD, Cheunsuang O, Stewart A, Morris R (2007). Morphology of inhibitory and excitatory interneurons in superficial laminae of the rat dorsal horn. J. Physiol..

[b26] Nagy GG, Al Ayyan M, Andrew D, Fukaya M, Watanabe M, Todd AJ (2004). Widespread expression of the AMPA receptor GluR2 subunit at glutamatergic synapses in the rat spinal cord and phosphorylation of GluR1 in response to noxious stimulation revealed with an antigen-unmasking method. J. Neurosci..

[b27] Naim M, Spike RC, Watt C, Shehab SA, Todd AJ (1997). Cells in laminae III and IV of the rat spinal cord that possess the neurokinin-1 receptor and have dorsally directed dendrites receive a major synaptic input from tachykinin-containing primary afferents. J. Neurosci..

[b28] Naim MM, Shehab SA, Todd AJ (1998). Cells in laminae III and IV of the rat spinal cord which possess the neurokinin-1 receptor receive monosynaptic input from myelinated primary afferents. Eur. J. Neurosci..

[b29] Nakaya Y, Kaneko T, Shigemoto R, Nakanishi S, Mizuno N (1994). Immunohistochemical localization of substance P receptor in the central nervous system of the adult rat. J. Comp. Neurol..

[b30] Oliveira AL, Hydling F, Olsson E, Shi T, Edwards RH, Fujiyama F, Kaneko T, Hokfelt T, Cullheim S, Meister B (2003). Cellular localization of three vesicular glutamate transporter mRNAs and proteins in rat spinal cord and dorsal root ganglia. Synapse.

[b31] Pezet S, Marchand F, D’Mello R, Grist J, Clark AK, Malcangio M, Dickenson AH, Williams RJ, McMahon SB (2008). Phosphatidylinositol 3-kinase is a key mediator of central sensitization in painful inflammatory conditions. J. Neurosci..

[b32] Polgár E, Campbell AD, MacIntyre LM, Watanabe M, Todd AJ (2007a). Phosphorylation of ERK in neurokinin 1 receptor-expressing neurons in laminae III and IV of the rat spinal dorsal horn following noxious stimulation. Mol. Pain.

[b33] Polgár E, Thomson S, Maxwell DJ, Al-Khater K, Todd AJ (2007b). A population of large neurons in laminae III and IV of the rat spinal cord have long dorsal dendrites and lack the neurokinin 1 receptor. Eur. J. Neurosci..

[b34] Polgár E, Watanabe M, Hartmann B, Grant SG, Todd AJ (2008a). Expression of AMPA receptor subunits at synapses in laminae I-III of the rodent spinal dorsal horn. Mol. Pain.

[b35] Polgár E, Al-Khater KM, Shehab S, Watanabe M, Todd AJ (2008b). Large projection neurons in lamina I of the rat spinal cord that lack the neurokinin 1 receptor are densely innervated by VGLUT2-containing axons and possess GluR4-containing AMPA receptors. J. Neurosci..

[b36] Ptak K, Burnet H, Blanchi B, Sieweke M, De Felipe C, Hunt SP, Monteau R, Hilaire G (2002). The murine neurokinin NK1 receptor gene contributes to the adult hypoxic facilitation of ventilation. Eur. J. Neurosci..

[b37] Puskár Z, Polgár E, Todd AJ (2001). A population of large lamina I projection neurons with selective inhibitory input in rat spinal cord. Neuroscience.

[b38] Rexed B (1952). The cytoarchitectonic organization of the spinal cord in the cat. J. Comp. Neurol..

[b39] Sakamoto H, Spike RC, Todd AJ (1999). Neurons in laminae III and IV of the rat spinal cord with the neurokinin-1 receptor receive few contacts from unmyelinated primary afferents which do not contain substance P. Neuroscience.

[b40] Schneider SP, Walker TM (2007). Morphology and electrophysiological properties of hamster spinal dorsal horn neurons that express VGLUT2 and enkephalin. J. Comp. Neurol..

[b41] Shi S, Hayashi Y, Esteban JA, Malinow R (2001). Subunit-specific rules governing AMPA receptor trafficking to synapses in hippocampal pyramidal neurons. Cell.

[b42] Todd AJ, Koerber HR, McMahon S, Koltzenburg M (2006). Neuroanatomical substrates of spinal nociception. Wall and Melzack’s Textbook of Pain.

[b43] Todd AJ, McGill MM, Shehab SA (2000). Neurokinin 1 receptor expression by neurons in laminae I, III and IV of the rat spinal dorsal horn that project to the brainstem. Eur. J. Neurosci..

[b44] Todd AJ, Hughes DI, Polgár E, Nagy GG, Mackie M, Ottersen OP, Maxwell DJ (2003). The expression of vesicular glutamate transporters VGLUT1 and VGLUT2 in neurochemically defined axonal populations in the rat spinal cord with emphasis on the dorsal horn. Eur. J. Neurosci..

[b45] Triller A, Seitanidou T, Franksson O, Korn H (1990). Size and shape of glycine receptor clusters in a central neuron exhibit a somatodendritic gradient. New Biol..

[b46] Watanabe M, Fukaya M, Sakimura K, Manabe T, Mishina M, Inoue Y (1998). Selective scarcity of NMDA receptor channel subunits in the stratum lucidum (mossy fibre-recipient layer) of the mouse hippocampal CA3 subfield. Eur. J. Neurosci..

[b47] Yoshimura M, Jessell T (1990). Amino acid-mediated EPSPs at primary afferent synapses with substantia gelatinosa neurones in the rat spinal cord. J. Physiol..

[b48] Yoshimura M, Nishi S (1992). Excitatory amino acid receptors involved in primary afferent-evoked polysynaptic EPSPs of substantia gelatinosa neurons in the adult rat spinal cord slice. Neurosci. Lett..

[b49] Zhu JJ, Esteban JA, Hayashi Y, Malinow R (2000). Postnatal synaptic potentiation: delivery of GluR4-containing AMPA receptors by spontaneous activity. Nat. Neurosci..

